# Therapeutic potential of hydrogen sulfide in osteoarthritis development

**DOI:** 10.3389/fphar.2024.1336693

**Published:** 2024-02-02

**Authors:** Yunjia Song, Siyu Wu, Rong Zhang, Qing Zhong, Xuanming Zhang, Xutao Sun

**Affiliations:** ^1^ Department of Pharmacology, School of Basic Medical Sciences, Heilongjiang University of Chinese Medicine, Harbin, China; ^2^ Department of Orthopedics, The Second Affiliated Hospital of Harbin Medical University, Harbin, China; ^3^ Department of Typhoid, School of Basic Medical Sciences, Heilongjiang University of Chinese Medicine, Harbin, China

**Keywords:** H_2_S, H_2_S donor, osteoarthritis, mechanism, therapeutic potential

## Abstract

The pathological mechanisms and treatments of osteoarthritis (OA) are critical topics in medical research. This paper reviews the regulatory mechanisms of hydrogen sulfide (H_2_S) in OA and the therapeutic potential of H_2_S donors. The review highlights the importance of changes in the endogenous H_2_S pathway in OA development and systematically elaborates on the role of H_2_S as a third gaseous transmitter that regulates inflammation, oxidative stress, and pain associated with OA. It also explains how H_2_S can lessen bone and joint inflammation by inhibiting leukocyte adhesion and migration, reducing pro-inflammatory mediators, and impeding the activation of key inflammatory pathways such as nuclear factor kappa B (NF-κB) and mitogen-activated protein kinase (MAPK). Additionally, H_2_S is shown to mitigate mitochondrial dysfunction and endoplasmic reticulum stress, and to modulate Nrf2, NF-κB, PI3K/Akt, and MAPK pathways, thereby decreasing oxidative stress-induced chondrocyte apoptosis. Moreover, H_2_S alleviates bone and joint pain through the activation of Kv7, K-ATP, and Nrf2/HO-1-NQO1 pathways. Recent developments have produced a variety of H_2_S donors, including sustained-release H_2_S donors, natural H_2_S donors, and synthetic H_2_S donors. Understanding the role of H_2_S in OA can lead to the discovery of new therapeutic targets, while innovative H_2_S donors offer promising new treatments for patients with OA.

## Graphical Abstract

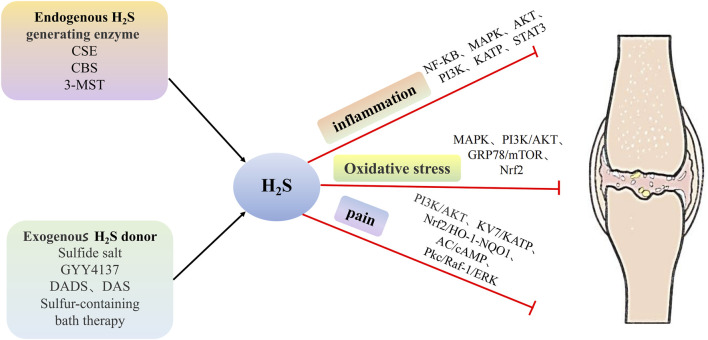

## 1 Introduction

Osteoarthritis (OA) is the most prevalent form of arthritis, particularly among the elderly population (Martel-Pelletier et al., 2016). Its primary characteristics include cartilage degeneration, bone calcification, and synovitis. Among these, the most prominent indicator is the deterioration of the hyaline articular cartilage (Loeser et al., 2012). According to the World Health Organization, OA has affected more than 50 billion people in recent years (Quicke et al., 2022). Currently, the primary treatment methods for OA encompass physical exercise and drug therapy. However, many patients require joint replacement surgery in the advanced stages of OA (Little and Hunter, 2013). This treatment option poses a significant financial burden, prompting active efforts to explore ways to prevent or offer alternative treatments for OA. Hydrogen sulfide (H_2_S) has been recognized as the third gas signaling molecule, following nitric oxide (NO) and carbon monoxide (CO) (Wang, 2002). It exerts a wide range of biological effects and plays crucial physiological and pathological roles in various human systems, including the cardiovascular, digestive, nervous, and reproductive systems (Wang, 2012). H_2_S also plays a pivotal role in regulating bone tissue function. H_2_S maintains bone anabolism and homeostasis through epigenetic differentiation of bone marrow mesenchymal stem cells (BMMSCs) (Behera et al., 2018a). Furthermore, numerous studies have indicated that a decline in the endogenous H_2_S content in the OA bone tissue. Supplementation with H_2_S can alleviate inflammation, oxidative stress, and pain responses in the progression of OA. This highlights the involvement of H_2_S in the development of OA and underscores its significant pathophysiological significance (Fox et al., 2012). H_2_S donors primarily include sulfide salts, GYY4137, organic sulfides such as diallyl disulfide (DADS) and diallyl sulfide (DAS) found in garlic. Experiments have substantiated the anti-inflammatory and protective effects of garlic on chondrocytes (Yang et al., 2017). Intra-articular administration of GYY4137 reduced cartilage destruction and oxidative damage (Vaamonde-García et al., 2020a). Bath therapy using hot sulfur water, an ancient treatment still in use today, has shown clear effectiveness in alleviating OA pain and symptoms (Verhagen et al., 2007). Mounting evidence supports the efficacy of exogenous H_2_S donors in OA treatment (Burguera et al., 2017). Currently, the pathogenesis of OA remains incompletely understood, and there is no definitive cure for the condition. However, accumulating evidence suggests the involvement of endogenous H_2_S in the OA process, and exogenous H_2_S donors hold promise as a potential treatment. Consequently, the pursuit of improved H_2_S donor replacement therapy represents a new trend. This review study comprehensively discusses the research progress of H_2_S donor therapy for OA, providing further insights into the regulatory effects and mechanisms of H_2_S on OA.

## 2 Generation of H_2_S

### 2.1 Generation of endogenous H_2_S

Traditionally considered a toxic gas, H_2_S has garnered recent attention due to studies recognizing it as the third gas signaling molecule, following endogenous NO and CO, playing a significant biological role (Zhao et al., 2015). The generation of endogenous H_2_S primarily occurs through enzyme-catalyzed and non-enzymatic pathways (Pan et al., 2017; Cao et al., 2019). Current research on H_2_S focuses primarily on the enzyme-catalyzed pathway (Liu et al., 2012; Wallace and Wang, 2015). Endogenous H_2_S is primarily composed of cystathionine-β-synthase (CBS), cystathionine-γ-lyase (CSE), and 3-mercapto pyruvate transferase (3-MST) (Singh et al., 2009). These enzymes responsible for H_2_S production in mammalian tissues exhibit tissue-specific expression (Pan et al., 2012). CSE is highly expressed in the heart, vascular endothelium, vascular smooth muscle, and kidney (Kimura, 2014; Polhemus and Lefer, 2014), while CBS is mainly expressed in BMMSCs, the central nervous system, ileum, kidneys, and islets (Behera et al., 2019). 3-MST has been identified in the brain, liver, kidney, and heart. Apart from the tissue specificity of these three enzymes, their distribution in the cell is also different (Pan et al., 2012; Donnarumma et al., 2017). Both CBS and CSE are cytoplasmic enzymes, while 3-MST is present in the cytoplasm and mitochondria of cells (Kamoun, 2004). In addition, endogenous H_2_S is also differentially expressed in bone tissue, with CSE, CBS, and 3-MST all being expressed in joint tissue. Fox et al. reported the detection of CBS and CSE in primary human articular chondrocytes (HACs) and mesenchymal progenitor cells (MPCs) undergoing the chondrogenic differentiation of HACs (Fox et al., 2012).

### 2.2 H_2_S donors classification in OA

H_2_S donors are compounds that degrade in response to specific stimuli, releasing H_2_S (Powell et al., 2018). These donors associated with osteoarthritis (OA) primarily include sulfide salts such as sodium sulfide (Na_2_S) and sodium hydrosulfide (NaHS); GYY4137; isoacyl isomers [e.g., allyl isothiocyanate (A-ITC)]; phenyl isomers [e.g., phenyl isothiocyanate (P-ITC)]; DAS, a component of garlic; DADS; diallyl trisulfide (DATS); polysulfides; ATB-346; and Lawesson reagent (LR reagent) (Table 1). Among these, Na_2_S and NaHS are the most common, as they rapidly release H_2_S without generating by-products. However, a drawback of sulfide salts is their uncontrollable H_2_S release. When the sulfide salts dissolves in the water buffer at 37°C and pH 7.4, it releases H_2_S, which evaporates rapidly and is lost in the storage solution (Zhao et al., 2015). Consequently, sulfide salts cannot accurately respond to enzymatic H_2_S production (Whiteman et al., 2010). GYY4137 represents a new water-soluble H_2_S donor. Compared with Na_2_S and NaHS, GYY4137 releases H_2_S at a slow and continuous rate. When GYY4137 was administered to rats, the plasma concentration of H_2_S peaked 30 min later and remained for 3 h. This indicates that GYY4137 releases H_2_S much more gradually than sulfide, allowing for sustained release (Hao et al., 2021). GYY4137, a H_2_S sustained-release donor with low cytotoxicity, is known for its relative stability. Upon administration of 200 μmol/kg GYY4137, the concentration of H_2_S in the liver and heart increases rapidly and is sustained for approximately 20 min. In the kidneys, H_2_S levels rise and remain elevated for a longer duration, eventually returning to normal levels after 2 h due to metabolic processes. In addition to these two common H_2_S donors, a variety of H_2_S donors have been developed. Natural H_2_S donors encompass A-ITC; P-ITC; and organic compounds such as DAS, DADS, and DATS found in garlic (Amagase, 2006; Magli et al., 2021). Qian et al. (2018) established that allicin, the primary active ingredient in garlic, possesses anti-inflammatory effects, which can contribute to the improvement of OA progression. The pharmacokinetic analysis of allicin in rats revealed that the peak concentration of allicin occurred between 30 and 60 min, and the average cumulative excretion of allicin in feces and urine reached 85.5% after 72 h (El-Saber Batiha et al., 2020). Recently, the role of H_2_S byproducts, specifically polysulfides, has gained attention as they have been found to possess biological activity, thereby highlighting the intricate chemistry of H_2_S. For instance, P*, a slow-releasing H_2_S and persulfide donor, has demonstrated anti-inflammatory and antioxidant effects in OA (Trummer et al., 2022). Synthetic H_2_S donors predominantly include ATB-346 and LR reagents. Among these, ATB-346 is a naproxen derivative that releases H_2_S, providing relief for OA-related pain (Dief et al., 2015). In preclinical studies, ATB-346 offers the advantage of lower gastrointestinal and cardiovascular toxicity when compared with naproxen (Caliendo et al., 2010). The LR reagent, commonly used in organic synthesis for sulfurization, releases H_2_S through spontaneous hydrolysis (Ozturk et al., 2007; Kang et al., 2017). Nicolau et al. (2013) discovered that the LR reagent, as an H_2_S donor, could prevent gastric damage induced by alendronate, a drug commonly used in the clinical treatment of bone diseases.

**TABLE 1:** Effects of H_2_S on the osteoarthritis.

Action	Mechanisms	Models	H_2_S donor (concentration)	Reference
Anti-inflammation	Inhibition of NF-κB pathway and NOS2, PEG2, COX2, IL-6 expression	CHs of OA patients	NaHS, GYY4137 (500 μM, 1000 μM)	Burguera et al. (2014)
	Inhibition of MAPK/PI3k/Akt pathway and IL-6, IL-8, RANTES, MMP-2, and MMP-14 expression	FLS of OA patients	NaHS (0.06–1 mM)	Sieghart et al. (2015)
	Inhibition of ERK/IκBα/NF-κB pathway and COX-2, MMP-13, and iNOS expression	CHs of OA patients	NaHS (0.06–1.5 mM)	Ha et al. (2015)
	Activation of KATP channel	C57bl/6 mice treated with kaolin/carrageenan	Na_2_S (50 μM)	Andruski et al. (2008)
	Inhibition of NF-κB pathway	MPCs and HAcs treated with IL-1β, IL-6, TNF-α and LPS	GYY4137 (200, 500 μM)	Fox et al. (2012)
	Inhibition of NF-κB pathway and COX-2 expression	Rats knee joint treated with MSU crystal	DAS (20 mM)	Lee et al. (2009)
	Inhibition of PI3K/Akt/NF-κB pathway	C57bl/6 mice DMM model	Allicin (10 mg/Kg)	Qian et al. (2018)
	Inhibition of c-Fos, NFATC1, DC-STAMP, OCSTAMP and NF-κB/STAT3 signaling pathway	LPS-induced mice model	DADS (40 mg/kg)	Yang et al. (2019)
Pro-inflammation	Activation of MAPK/NF-κB pathway and IL-6 level	FLS of OA patients	NaHS (above 0.5 mM)	Kloesch et al. (2012)
Alleviate pain	Inhibition of AC/cAMP and PKC/Raf-1/ERK pathway and cGRP expression	Opioid withdrawal-induced pain sensitization rat model	NaHS (1.4 μg)	Yang et al. (2014)
	Activation of DRG and AMG expression	C57BL/6 mice treated with sodium iodoacetate	GYY4137 (0.4 mg/kg), DADS (3 mg/kg)	Batallé et al. (2021)
	Activation of Kv7/KATP channel and Nrf2/HO-1-NQO1 pathway	C57BL/6J mice treated with plantar injection of CFA	DADS (70, 100, 200 μM/kg), PITC (23.2, 29 µM/kg)	Porta et al. (2021)
	Inhibition of NOS2, PI3K, p-AKT level	Intraarticular injection of sodium iodoacetate-induced OA pain in C57BL/6 mice	DADS (200 μM/kg), GYY4137 (32 μM/kg)	Batallé et al. (2021)
	Activation of KATP channel and inhibition of NOS2, PI3K, p-AKT.	Intraarticular injection of sodium iodoacetate-induced OA pain in C57BL/6 mice	AITC (4.4 μM/kg), PITC (13.3 μM/kg)	Batallé et al. (2023)
Relieve mitochondrial dysfunction	Activation of mito-SOD-2 and Mfn-2 and inhibition of cytc	MTC3T3-E1 in osteoblasts treated with Hcy	NaHS (30 μM, 300 μM)	Zhai et al. (2019)
	Inhibition of MAPK signaling pathway	CHs treated with IL-1β	NaHS (1 mM)	Wang et al. (2021)
	Inhibition of Akt and ERK 1/2 signaling pathways	MPCs and HAcs treated with H_2_O_2_, SIN-1 and 4-HNE	GYY4137 (200, 500 μM)	Fox et al. (2012)
	Activation of Nrf-2, HO-1 and BCl2. Inhibition of MAPK signaling pathway and caspase 3, Bax expression	C28I2 human chondrocytes treated with IL-1β	DADS (1, 5, 10 μM)	Hosseinzadeh et al. (2017)
Antioxidant	Inhibition of NF-κB pathway and ROS, MDA. Activation of CAT, GPx1, GPx3, GPx4 and SOD1	HADSCs treated with IL-1β	DADS (1 μM)	Bahrampour Juybari et al. (2018)
	Activation of Nrf-2/HO-1-NQO1 and MAPK/PI3k/Akt signaling pathway	Chondrocyte cells isolated from OA patients	NaHS (0.5 mM), P* (0.5 mM)	Trummer et al. (2021)
	Inhibition of GRP78/mTOR signaling pathway	CHs of rats hip joints treated with TBHP	GYY4137 (100 μM)	Wu et al. (2021)
	Activation of Nrf-2, NQO1 and HO-1 and inhibition of MMP13, NOS2 and COX-2 expression	Wistar rat ACLT model	GYY4137 (200 μM)	Vaamonde-Garcia et al. (2020a)
	Activation of Nrf-2, HO-1 and BCl2	C28I2 human chondrocytes treated with IL-1β	DADS (1, 5, 10 μM)	Hosseinzadeh et al. (2017)

Abbreviation: CHs, chondrocytes; OA, osteoarthritis; FLS, fibroblast-like synoviocytes; DMM, destabilization of the medial meniscus; CFA, complete Freund’s adjuvant; Hcy, homocysteine; MPCs, mesenchymal progenitor cells; HAcs, human articular chondrocytes; HADSCs, human adipose-derived mesenchymal stem cells; ACLT, anterior cruciate ligament transection.

Furthermore, apart from the aforementioned H_2_S donors associated with OA, other H_2_S donors have been investigated in various diseases. Although these studies did not specifically mention OA, they offer valuable insights for future research directions. 1,2-dithioether-3-thiophenone (DTT) is believed to play a central role in the liberation of H_2_S, which is generated through the hydrolysis of DTTs (Qandil, 2012). DTTs are commonly employed in the preparation of combination medications for H_2_S donation, such as S-aspirin and S-diclofenac (Zhao et al., 2015). DM-22 serves as a thiol-activated H_2_S donor with sustained-release properties. The release of H_2_S by DM-22 exhibits sustained kinetics and lacks cytotoxicity (Rapposelli et al., 2017). SDSS, a donor derived from the synthesis of Danshensu (DSS) and H_2_S, represents a synthetic H_2_S donor within traditional Chinese medicine (Hao et al., 2021). SDSS demonstrates significant antioxidant effects. However, there is currently a dearth of research investigating the relationship between SDSS and OA, which may offer novel perspectives for OA research. In addition, recent advancements in the field have led to the synthesis of a series of H_2_S delivery compounds that specifically target mitochondria, such as AP39. Szczesny et al. (2014) has demonstrated that AP39 serves as an antioxidant and cell protective agent during oxidative stress. Notably, AP39 exhibits beneficial effects within a concentration range of 30–100 nM, which is significantly lower than that of NaSH, LR, Na_2_S, and GYY4137, suggesting that AP39 is more efficacious than H_2_S donors like sulfide salts. The development of H_2_S delivery compounds that specifically target mitochondria represents a novel and valuable research tool for investigating the impact of H_2_S on cellular bioenergy. Moreover, these compounds hold promise for the development of new therapeutic approaches for OA.

In addition, the biological effects of H_2_S are often mediated indirectly through processes such as persulfidation or interaction with metalloproteins. It is widely accepted that the primary mechanism of H_2_S signaling involves the reaction between H_2_S and protein sulfhydryl groups, resulting in the formation of persulfides (protein persulfidation). This conversion, specifically from cysteine sulfate (Cys-S-) to persulfides (Cys-S-S-), is considered crucial for H_2_S to exert its diverse range of biological effects, including but not limited to anti-inflammatory response, anti-apoptosis and anti-oxidative stress, as well as cell survival, cell proliferation, metabolism, and mitochondrial function (Song et al., 2023). Furthermore, H_2_S can exert its biological functions through extensive interactions with metalloproteins. Metalloproteins play crucial roles in the metabolic pathway of oxygen within the body, encompassing the transportation and storage of O_2_, cellular respiration, and the maintenance of redox homeostasis by eliminating reactive oxygen species (ROS). The coordination and redox reactions involving metal centers serve as the primary mechanisms by which H_2_S modulates fundamental cellular processes. The reduction of high-valent metal centers by H_2_S not only affects metalloprotein functions, but also leads to radical formation and subsequent polysulfide formation. These processes may play a role in cellular protection against oxidative stress and redox signaling. Additionally, recent research has highlighted the potential of H_2_S as substrates for mitochondrial energy production and their cytoprotective capacity, involving metalloproteins (Domán et al., 2023). Consequently, gaining a comprehensive understanding of the molecular mechanism of H_2_S is crucial for the development of H_2_S-related drugs.

## 3 Overview of osteoarthritis

OA is a degenerative joint disease often associated with joint pain, deformity, and dysfunction, particularly prevalent among the elderly population (Hao et al., 2021). According to statistics indicate that more than 50 billion people worldwide have been affected by OA (Quicke et al., 2022). Although OA can occur in any joint, it primarily affects the knees, hands, hips, and spine (Favero et al., 2022; Hall et al., 2022). This condition also leads to changes in other joint tissues such as ligaments, synovium, and subchondral bone (Brandt et al., 2008). OA is a major factor limiting the daily activities of the elderly (Xia et al., 2014). Various risk factors affecting OA have been identified, including individual factors (sex, age, aging, and obesity) and joint-specific factors (injury, dislocation, and abnormal joint load) (Palazzo et al., 2016). While the complete etiology of OA remains unclear, its main pathological features include focal loss of articular cartilage within synovial joints, bone calcification, along with bone hypertrophy (such as osteophyte growth and subchondral osteosclerosis), and thickening of the joint capsule (Felson et al., 2000). Clinical symptoms of OA encompass limited joint function, stiffness, pain, and in severe cases, disability (Yao et al., 2023). Formerly considered a straightforward disease of articular cartilage wear (Abramoff and Caldera, 2020), OA is now understood to be a more intricate pathological process involving inflammatory and metabolic factors (Musumeci et al., 2015; Mobasheri and Batt, 2016). OA exhibits a conspicuous chronic inflammatory state, characterized by the presence of local inflammatory molecules such as IL-1β, TNF-α, IL-6, nitric oxide (NO), and PGE2. These molecules play a crucial role in promoting the progression of OA, particularly in its early stages, ultimately leading to the establishment of chronic inflammation. As the disease advances, the sustained release of pro-inflammatory cytokines triggers the continuous production of proteases, which in turn results in extensive degradation and loss of the extracellular matrix. Consequently, significant stromal damage occurs, leading to the induction of chondrocyte apoptosis (Yunus et al., 2020). Consequently, *in vitro* models of OA often involve the induction of chondrocytes using IL-1β, TNF-α, IL-6, or LPS (Goldring and Otero, 2011; Kapoor et al., 2011; Nedunchezhiyan et al., 2022). Articular cartilage primarily comprises extracellular matrix (ECM) and chondrocytes (Luo et al., 2017). Chondrocytes (CHs) represent the only cell type present in cartilage. Prolonged excessive mechanical stress on the body leads to cartilage damage, causing chondrocytes to aggregate in the affected area. This results in increased growth factors in the ECM, leading to cell proliferation and ECM synthesis. As the degree of articular cartilage injury worsens, chondrocyte apoptosis, ECM synthesis, and eventually articular cartilage degeneration occur (Suri and Walsh, 2012). With cartilage erosion, the two bones rub against each other, further exacerbating OA. The heightened expression of matrix-degrading enzymes contained within the ECM, such as matrix metalloproteinases (MMP), a disintegrin and metalloproteinase with thrombospondin motifs (ADAMTS), as well as the breakdown of type II collagen α1 and aggregator glycan, are among the most well-known mechanisms of OA (Hollander et al., 1995; Tao et al., 2021). Several signaling pathways, including Wnt, mitogen-activated protein kinase (MAPK), and nuclear factor kappa B (NF-κB) pathways, have been associated with OA (Deshmukh et al., 2019; Li et al., 2020; Ostojic et al., 2021). In addition to chondrocytes, fibroblast-like synoviocytes (FLS) also contribute to OA progression (Chen et al., 2022). The synovial membrane is a connective tissue covering the inner surface of the joint capsule. It produces synovial fluid, which reduces joint friction, minimizes cartilage loss, and ensures the metabolism and nutrition of articular cartilage (Pap et al., 2020). Patients with OA are highly likely to experience synovitis, which is correlated with joint pain and dysfunction in OA development (Sowers et al., 2011).

## 4 Endogenous H_2_S and OA

A substantial body of evidence indicates a close association between endogenous H_2_S and bone development. Burguera et al. (2020) conducted a quantitative reverse transcription–polymerase chain reaction assay and Western blot analysis on cartilage isolated from both normal individuals and patients with OA. Their findings revealed that, in the OA group, the expression levels of 3-MST mRNA and protein, as well as H_2_S content, were significantly reduced compared with the normal group. Interestingly, no significant changes were observed in CBS and CSE levels. This suggests that the notable reduction of 3-MST in the OA cartilage may lead to diminished H_2_S biosynthesis in this tissue, potentially contributing to OA progression (Burguera et al., 2020).

Fox et al. (2012) discovered that both CSE and CBS could be detected in MPCs isolated from cartilage in patients with OA, with CBS exerting a more substantial influence on H_2_S synthesis than CSE. Furthermore, upon induction of MPCs and HACs by pro-inflammatory cytokines tumor necrosis factor alpha (TNF-α), interleukin (IL)-1, and IL-6, the expression and activity of CSE were significantly elevated when compared with those of CBS. Treatment of chondrocytes induced by pro-inflammatory cytokines with p38, NF-κB, and ERK1/2 inhibitors resulted in a marked reduction of H_2_S synthesis, indicating that pro-inflammatory cytokines regulate H_2_S synthesis by modulating CSE expression through the p38/ERK/NF-κB signaling pathway. Additionally, both MPCs and HACs exhibited cell death in response to SIN-1, H_2_O_2_, and 4-HNE. Treatment with L-cysteine significantly mitigated oxidative stress-induced cell death in MPCs, an effect reversed by CSE and CBS inhibitors PAG and AOAA, respectively. These findings suggest that endogenous H_2_S may represent a novel protective mechanism, with both CSE and CBS playing a role in shielding cells from oxidative stress. Wei et al. (2023) observed downregulated expression of CSE in cartilage tissue samples from patients with OA compared with that in normal cartilage tissue samples. Furthermore, they established an OA rat model through anterior cruciate ligament transection (ACLT) surgery, revealing a higher abundance of osteophytes in the ACLT group than in the normal control group. However, when the CSE-overexpressed lentivirus was injected into the joint cavity of the ACLT group, osteophyte formation was notably reduced. In in vitro experiments, rat chondrocytes were induced by IL-1β to create an OA model. Following treatment with CSE-overexpressed lentivirus, the expression of MMP3 and MMP13 was downregulated, while the expression of aggregation protein, collagen 2, and Sox9 was upregulated. Furthermore, phosphorylation levels of the NF-κB pathway proteins IKK, p65, and IκB were reduced. Conversely, lowering CSE expression led to downregulated mRNA expression of Aggrecan, collagen 2, and Sox 9, accompanied by increased IKK, p65, and IκB phosphorylation levels. These results demonstrate that CSE expression can mitigate chondrocyte degradation, restore bone remodeling, and alleviate OA inflammation and cartilage deterioration by inhibiting the NF-κB pathway. One of the prominent features of OA is the formation and deposition of calcium-containing crystals in joint tissues, with endogenous H_2_S playing a role in regulating bone mineralization. Nasi et al. (2020) established a negative correlation between the expression level of 3-MST in human cartilage and bone tissue calcification, as well as OA severity. They also noted a significant reduction in 3-MST expression levels after stimulation with hydroxyapatite crystals. In a meniscectomy mouse model, it was observed that 3-MST in the knee cartilage of OA-afflicted mice with meniscectomy was diminished compared with healthy knee joints undergoing sham surgery. Additionally, meniscectomy in 3-MST^−/−^ mice resulted in greater joint calcification, proteoglycan loss, and OA severity than meniscectomy in wild-type mice. Further *in vitro* studies demonstrated that the use of 3-MST inhibitors significantly reduced H_2_S production in chondrocytes, leading to chondrocyte calcification and heightened IL-6 secretion. These findings suggest that H_2_S produced by 3-MST may protect against joint calcification and OA. Enhancing H_2_S production in chondrocytes may be a potential drug target for OA treatment. CBS is primarily found in BMMSCs, and Behera et al. (2018b) have demonstrated that H_2_S maintains bone anabolism and homeostasis through epigenetic differentiation of BMMSCs. Sui et al. (2022) established an OA rat model through ACLT surgery, observing swelling and limited activity in the rat knee joints. Following treatment with the OA drug rutin, the knee joint swelling persisted, but normal movement was restored. In comparison to normal cartilage, the expression of ROCK1 and ROCK2 in the OA cartilage of rats was significantly increased, while the expression of CBS was downregulated. In addition, in an *in vitro* OA model using the mouse chondrocyte cell line ATDC5 induced by lipopolysaccharide (LPS), the expression of the pro-inflammatory cytokines inducible NO synthase (iNOS), COX-2, TNF-α, and MMP13 was upregulated, while the expression of Col II and Aggrecan was downregulated. After treatment with rutin, a significant decrease was observed in the expression of pro-inflammatory cytokines, as well as in the RhoA downstream effectors ROCK1 and ROCK2. By contrast, a substantial increase was observed in the expression of Col II and Aggrecan. When comparing this with the LPS-induced group, the expression of pro-inflammatory cytokines and ROCK1 and ROCK2 was upregulated, and the expression of Col II and Aggrecan was downregulated after CBS knockdown. However, in LPS-induced chondrocytes treated with the CBS overexpression vector, the expression of pro-inflammatory cytokines was downregulated, that of Col II and Aggrecan was significantly upregulated, and that of ROCK1 and ROCK2 was downregulated. In summary, rutin negatively regulates the RhoA/ROCK signaling pathway by upregulating CBS expression to inhibit the OA inflammatory response and upregulating CBS can influence OA progression. To summarize, CSE, CBS, and 3-MST play crucial roles in regulating the physiological and pathological processes of OA. H_2_S may represent a novel endogenous mechanism in OA, presenting potential opportunities for the treatment and intervention of this condition.

## 5 H_2_S regulates OA

### 5.1 The inflammation regulation of H_2_S in OA

**FIGURE 1:**
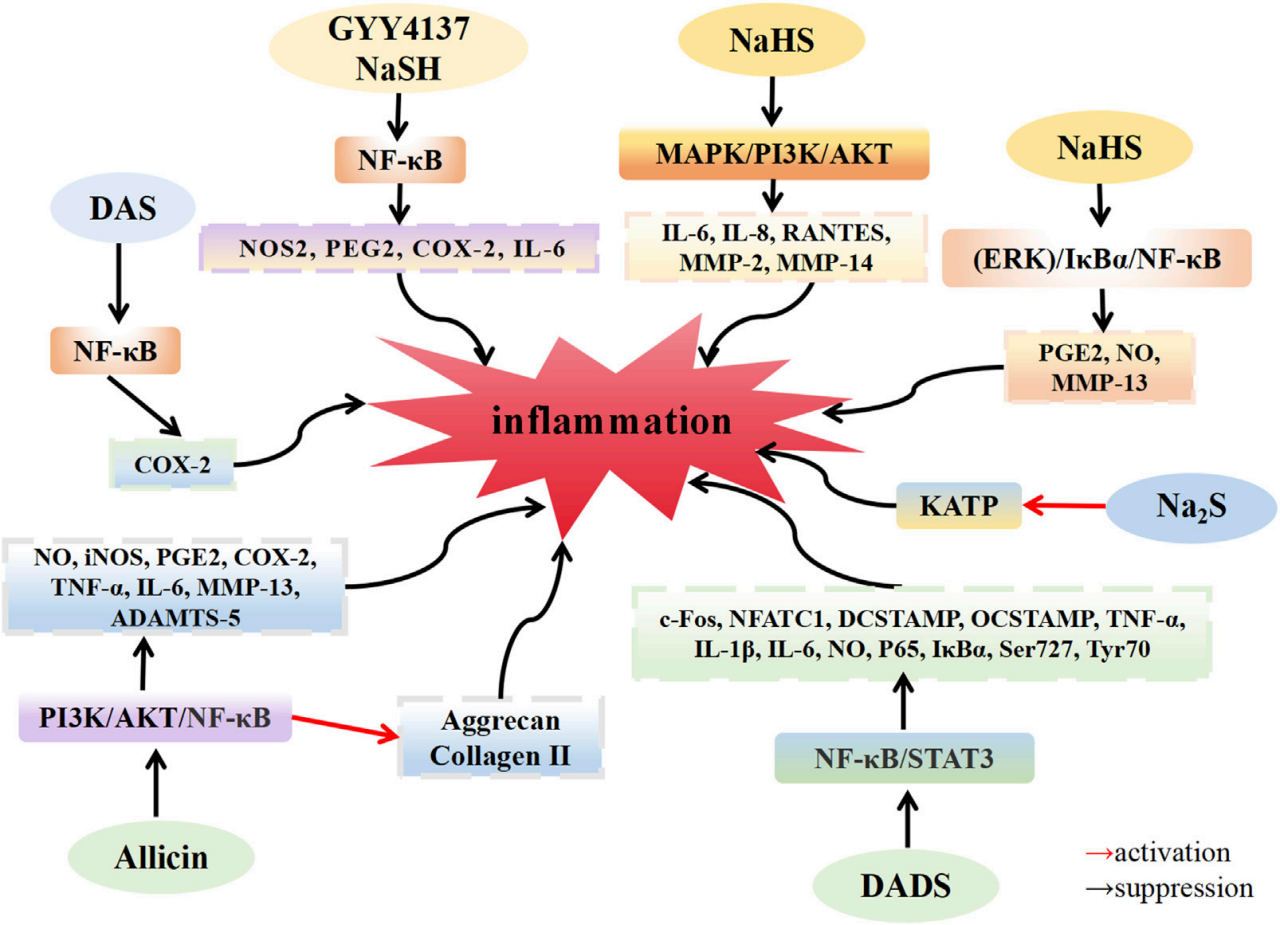
H_2_S inhibit the inflammation in osteoarthritis. H_2_S donors: GYY4137, NaHS, Na_2_S, DAS, DADS, Allicin.

Inflammatory factors play a significant role in the development of OA. H_2_S is a key regulator of inflammation, affecting various critical mechanisms and pathways, which are as follows: (1) inhibiting leukocyte adhesion and migration, (2) reducing the expression of pro-inflammatory mediators such as NO, PGE2 and so on, (3) blocking the activation of classical inflammatory signaling pathways, such as NF-κB, MAPK, and PI3K/AKT. Notably, the anti-inflammatory effect of H_2_S in the OA model depends not only on the concentration of H_2_S but also on the timing of administration (Figure 1).

Muniraj et al. (2017) discovered that the levels of H_2_S in the plasma of patients with OA were significantly higher than those in healthy individuals, highlighting the presence of H_2_S in the body. This suggests that H_2_S could be a promising target for OA treatment. Key features of joint inflammation involve alterations in tissue blood flow, increased vascular permeability, and the accumulation of white blood cells within joint tissues. The entry of white blood cells into joint tissues results from a multistep complex interaction between these cells and activated endothelium (Gál et al., 2005). H_2_S may be involved in this interaction between white blood cells and endothelium. Andruski et al. (2008) established an acute arthritis model in C57BL/6 mice by injecting kaolin/carrageenan into the joints. Compared with normal mice, OA mice exhibited slower rolling speeds and increased adhesion of white blood cells in the knee microvessels. However, after administering H_2_S donor Na_2_S (50 μM) into the knee joint, white blood cell rolling speeds increased, and adhesion decreased in OA mice. Notably, the effects of Na_2_S on rolling speed and adhesion were completely reversed by glibenclamide, a potassium channel inhibitor. These results indicate that H_2_S inhibits leukocyte transport and reduces leukocyte adhesion by activating KATP channels, emphasizing its significant anti-inflammatory effect in this arthritis model. Overactivation of osteoclasts and excessive bone resorption can lead to various lytic bone diseases, including OA (Quan et al., 2015). In the presence of RANKL, NF-κB translocates to the nucleus, activating the osteoclast regulator NFATc1 promoter to promote the expression of NFATc1 (Tomomura et al., 2015). Yang et al. (2019) found that under RANKL induction, mouse bone marrow macrophages (BMMs) formed osteoclasts. After treatment with DADS, the expression of osteoclast marker genes, c-Fos, NFATC1, DC-STAMP, and OC-STAMP, were downregulated. Furthermore, when BMMs were induced by LPS to produce osteoclasts, DADS treatment resulted in the downregulated expression of TNF-α, IL-1β, IL-6, and NO, along with decreased phosphorylation levels of Ser727 and Tyr705 of NF-κB p65, IκB-α, and STAT3. These findings suggest that DADS inhibits RANKL and LPS-induced osteoclast formation by inhibiting the NF-κB and STAT3 signaling pathways. DADS also reduces osteoclast-related pro-inflammatory cytokines. Vaamonde-Garcia et al. (2020b) established an OA rat model through the transection of the medial collateral ligament of the left joint and resection of the medial meniscus of the left joint. Following treatment with GYY4137 (200 μM), the locomotor activity of rats exhibited a significant improvement, and the expression of pro-inflammatory mediators COX-2, NOS2 and MMP13 were downregulated. These results indicate that the H_2_S donor GYY4137 can lower the expression of pro-inflammatory mediators and alleviate OA symptoms. Lee et al. (2009) established an *in vitro* OA model by injecting monosodium urate (MSU) crystals into the knee joint of rats, where they observed an increase in COX-2 expression in OA rats compared with that in healthy rats. However, COX-2 expression was significantly inhibited in OA rats after injecting H_2_S donor DAS (20 mM). Furthermore, when the HIG-82 synovial cell line was induced with MSU crystals in an *in vitro* OA model, COX-2 expression increased. However, after co-incubation with different concentrations of H_2_S donor DAS, DAS inhibited COX-2 upregulation in a dose-dependent manner. In chondrocytes and synovial tissue samples from patients with OA induced by MSU crystals and IL-1β, COX-2 expression was significantly upregulated, along with activation of the NF-κB pathway. However, DAS treatment led to significant downregulation of COX-2 expression and inhibition of the NF-κB pathway. These findings suggest that DAS prevents IL-1β and MSU crystal-induced upregulation of COX-2 in synovial cells and chondrocytes, potentially ameliorating OA inflammation by inhibiting the NF-κB pathway. The anti-inflammatory effects of DAS hold therapeutic promise for OA. Qian et al. (2018) established an OA model in C57BL/6 male mice with destabilization of the medial meniscus. Comparing these mice to sham-operated mice, the cartilage chyme of OA mice exhibited deteriorated characteristics. However, after allicin injection, the cartilage surface of the mice became smoother, the nuclear translocation of p65 was reduced, and the phosphorylation levels of PI3K and AKT decreased. Subsequent *in vitro* experiments revealed upregulated expression of the pro-inflammatory cytokines NO, iNOS, PGE2, COX-2, TNF-α, IL-6, MMP-13, and ADAMTS-5, along with inhibited the synthesis of type II collagen and agglomerin in the knee cartilage tissue samples of C57BL/6 mice induced by IL-1β. Upon treatment with 5 μM and 10 µM allicin, allicin inhibited proinflammatory cytokine production and restored type II collagen and agglomerin synthesis in a dose-dependent manner. In addition, IL-1β stimulation significantly decreased the expression of IκB-α in the cytoplasm, increased the expression of p65 in the nucleus, and increased the phosphorylation levels of PI3K and AKT. However, allicin treatment inhibited these effects in a dose-dependent manner, aligning with *in vivo* results. These results suggest that allicin can serve as an anti-inflammatory agent in OA treatment, inhibiting the expression of IL-1β-induced inflammatory mediators by suppressing the PI3K/Akt/NF-κB pathway in mouse chondrocytes. Dehghani et al. (2018) reported that the concentration of proinflammatory adipocytokines in the serum of overweight female patients with OA decreased after a 12-week allicin regimen, indicating the potential of allicin as a therapeutic agent for patients with OA in clinical settings. Burguera et al. (2014) reported that, after IL-1β induction in human CHs isolated from patients with OA, the expression of the pro-inflammatory mediators NOS2, PEG2, COX2, and IL-6 was upregulated, and NF-κB p65 translocated to the nucleus. However, treatment with 500 μM and 1000 µM NaSH and GYY4137 led to downregulation of NOS2, PEG2, COX2, and IL-6 expression, along with a reduction in NF-κB p65 translocation to the nucleus. These experimental findings demonstrate that exogenous H_2_S supplementation can modulate the expression of related genes in the pathogenesis and progression of OA, countering the pro-inflammatory signal of IL-1β, and contributing to cartilage destruction by inhibiting NF-κB activation. Sieghart et al. (2015) discovered that following induction by IL-1β, FLS isolated from the synovial tissue of patients with OA exhibited upregulated expression of IL-6, IL-8, RANTES, MMP-2, and MMP-14, which are associated with the progression of OA. MAPK kinase phosphorylation increased and induced changes in the microcluster structure, leading to lining layer proliferation. Subsequent NaSH (1000 µM) treatment resulted in the downregulation of IL-6, IL-8, RANTES, MMP-2, and MMP-14 expression levels, along with decreased phosphorylation levels of MAPK, while Akt1/2 phosphorylation levels increased. These findings suggest that exogenous H_2_S may inhibit IL-1β stimulation and alleviate OA inflammation by selectively modulating the MAPK kinase and a portion of the PI3K/Akt pathway. Ha et al. (2015) observed that upon IL-1β induction of cartilage tissue samples from patients with OA, IL-1β promoted the production of inflammatory mediators PGE2, NO, and MMP-13. However, following treatment with various concentrations of NaSH, significant inhibition of inflammatory mediators occurred at 0.3 mM NaSH, while mild inhibition was observed at 1.5 mM NaSH. The expression of COX-2, MMP-13, and iNOS is pivotal for chondrocyte degradation. At 0.3 mM NaSH concentration, NaHS suppressed COX-2, MMP-13, and iNOS expression, whereas 1.5 mM NaSH showed minimal anti-inflammatory effect. This suggests that H_2_S does not exert its anti-inflammatory effects in a strictly dose-dependent manner, and high concentrations of H_2_S may not possess anti-inflammatory properties. NaSH reduced NF-κB p65, IκBα, and ERK1/2 phosphorylation levels compared with non-IL-1β induction groups. The ERK pathway inhibitor PD98059 completely reversed the inflammatory effect of H_2_S. These results imply that H_2_S shields chondrocytes by inhibiting the IL-1β-induced (ERK)/IκBα/NF-κB signaling pathway.

Extensive research has demonstrated that the role of H_2_S in inflammation is dependent on its concentration, with high and low concentrations exerting opposing effects on chondrocytes and synovial cells, namely, pro-inflammatory and anti-inflammatory, respectively (Blachier et al., 2019). Kloesch et al. (2012) investigated human FLS isolated from the synovial tissue of patients with OA. After treatment with 1.0 mM NaSH, the expression of the pro-inflammatory mediators IL-6, IL-8, and COX-2 was upregulated, and the phosphorylation of ERK1/2 increased. Inhibitors of p38MAPK (SB203580), ERK1/2 (U0126), and NF-κB (BAY11-7082) were employed to suppress the production of pro-inflammatory mediators. These results indicate that high concentrations of H_2_S (above 0.5 mM) exacerbate OA inflammation by activating the MAPK/NF-κB pathway. Kloesch et al. (2010) also discovered that *in vitro* cultured FLS spontaneously produced a substantial amount of IL-6 and IL-8. FLSs at a low concentration of 0.125 mM NaHS downregulated the expression of IL-6, whereas inhibition disappeared when the concentration of NaHS exceeded 0.125 mM. This suggests that low concentrations of H_2_S exhibit a notable anti-inflammatory effect, while high concentrations promote the development of inflammation. Li et al. (2013) established an acute arthritis model by injecting complete Freund’s adjuvant (CFA) into CD1 mice. After 24 h of CFA injection, the mice exhibited swelling in the knee joint. When GYY4137 was injected 1 h before CFA administration, the knee joint swelling in the mice was more severe. However, the expression levels of myeloperoxidase, N-acetyl-beta-D-glucosaminidase (NAG), TNF-α, IL-1β, IL-6, and IL-8 were downregulated by GYY4137 injection 6 h after CFA administration. These results indicate that the proinflammatory or anti-inflammatory effect of H_2_S in the OA model depends not only on the concentration of H_2_S but also on the timing of administration. In conclusion, varying concentrations of H_2_S play distinct roles in modulating the inflammatory response, with low concentrations exhibiting anti-inflammatory effects and high concentrations promoting inflammation.

### 5.2 The redox regulation of H_2_S in OA

**FIGURE 2:**
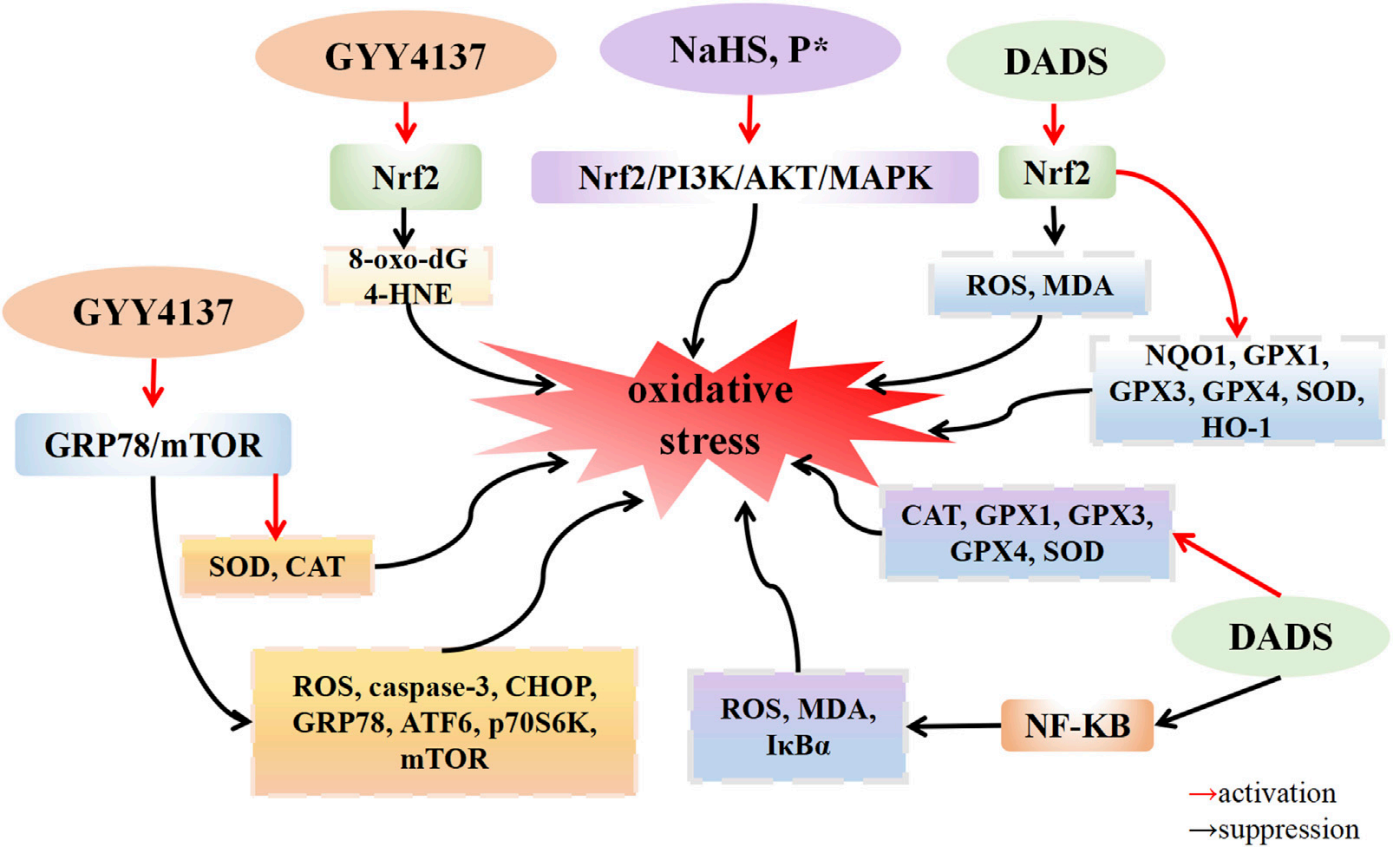
H_2_S suppress oxidative stress in osteoarthritis. H_2_S donors: cc, P*, DADS.

H_2_S possesses the potential to serve as an endogenous medium for the purpose of limiting the detrimental effects caused by free radicals (Whiteman et al., 2004). The presence of H_2_S is crucial in maintaining the balance of Redox, thereby exerting an influence on the Redox state of cells. H_2_S exhibits robust reducing properties, enabling it to directly interact with various oxidative stressors, encompassing superoxide radical anions, hydrogen peroxide, and peroxynitrite (ONOO-). Furthermore, H_2_S has the ability to augment the activity of superoxide dismutase (SOD), a key component of the cellular antioxidant defense system, and enhance the clearance of ROS (Mitsuhashi et al., 2005). Oxidative stress is the primary cause of chondrocyte apoptosis in OA. A growing body of evidence indicates that the increase in reactive oxygen species (ROS) within OA leads to oxidative stress, and ROS is closely linked with the inflammatory response. Nasi et al. (2020) discovered that H_2_O_2_-induced ATDC5 cells exhibited a downregulated expression of 3-MST expression and an upregulated expression of the calcification genes Ak1 and Ak5. This suggests that when 3-MST is inhibited, H_2_O_2_ intensifies chondrocyte calcification. The application of the ROS scavenger NAC reduced the degree of cartilage calcification. Experimental results demonstrate that ROS, such as H_2_O_2_, decrease 3-MST expression, resulting in reduced endogenous H_2_S production. This leads to chondrocyte calcification, thereby promoting OA progression. This suggests that oxidative stress downregulates endogenous hydrogen sulfide 3-MST, accelerating cartilage calcification. Vaamonde-Garcia et al. (2020a) established an OA model by transecting the medial collateral ligament and excising the medial meniscus of the left joint in rats. In the GYY4137 treatment group, expression levels of 8-oxo-dG and 4-HNE, which are related to DNA damage, were downregulated, while Nrf2 gene expression was upregulated compared with that in the model group. These results indicate that GYY4137 could protect the cartilage and reduce oxidative damage to chondrocytes by upregulating the expression of Nrf2. Because of increased inflammation and oxidative stress in the OA microenvironment, the differentiation of mesenchymal stem cells (MSCs) into chondrocytes is hindered. Bahrampour Juybari et al. (2018) reported that IL-1β induced human adipose-derived mesenchymal stem cells (hADSCs) to upregulate the expression levels of ROS and membrane lipid peroxide malondialdehyde (MDA), while downregulating the expression of the cartilage markers COMP, COL2a, SOX9, and aggrecan. This led to an increased phosphorylation level of IκBα. After H_2_S donor DADS treated hADSCs induced by IL-1β, the expression of ROS and MDA was downregulated, the expression of the antioxidant enzymes catalase (CAT), GPx1, GPx3, GPx4, and SOD1 was upregulated, and the phosphorylation level of IκB-α was decreased. These findings suggest that DADS prevents cartilage destruction and dyschondrogenesis by inhibiting the activation of the NF-κB signaling pathway. Oxidative stress can trigger apoptosis through the mediation of mitochondrial dysfunction and endoplasmic reticulum stress (Tang et al., 2017). Wu et al. (2021) discovered that CHs isolated from both hip joints of rats, when induced by tert-butylhydroxide (TBHP), undergo apoptosis, leading to an upregulation of ROS expression in both the cytoplasm and mitochondria. This inhibits the activities of superoxide dismutase (SOD) and CAT in the mitochondria. Compared with the TBHP induction group, treatment with GYY4137 (100 μM) or the antioxidant NAC resulted in the downregulation of cytoplasmic and mitochondrial ROS, enhanced activities of SOD and CAT in the mitochondria, and inhibited 1-methyl-4-phenyl-1,2,3,6-tetrahydropyridine (mPTP) opening. Additionally, after GYY4137 treatment, the expression of the apoptosis protein Caspase-3 was also downregulated. Compared with the control group, the expression of CHOP, GRP78, and ATF6 in the TBHP induction group was upregulated. However, after further knocking down GRP78, the expression of CHOP, GRP78, and ATF6 in the GYY4137 treatment group was downregulated. The phosphorylation levels of P70S6K and mTOR were upregulated in the TBHP group compared with the control group. Nevertheless, after treatment with GYY4137, GRP78 siRNA, or the Toll-like receptor inhibitor chloroquine, phosphorylation levels of P70S6K and mTOR were downregulated compared with the TBHP group. These results indicate that GYY4137 alleviates endoplasmic reticulum stress and reduces chondrocyte apoptosis by inhibiting the GRP78/mTOR pathway. Hosseinzadeh et al. (2017) observed that, after the induction of IL-1β, C28I2 human chondrocytes upregulated the expression levels of intracellular ROS and MDA, and downregulated the expression of the detoxase genes NQO1 and GSTP1, and the antioxidant genes CAT, GPx1, GPx3, and GPx4. Following DADS treatment, the expression of ROS and MDA was downregulated, and the expression of NQO1 and GSTP1, CAT, GPx1, GPx3, and GPx4 genes was upregulated. Compared with the IL-1β group, the DADS group demonstrated upregulated expression levels of the nuclear protein HO-1 and Nrf2, while downregulating the expression levels of the mitochondrial apoptosis markers Bax/Bcl-2 and caspase-3. Additionally, it downregulated the IL-1β-induced phosphorylation levels of JNK and p38. Experimental results showed that DADS alleviated IL-1β-induced oxidative stress and chondrocyte apoptosis by increasing Nrf2 nuclear translocation and upregulated the expression of the detoxification enzyme and the antioxidant enzyme, thereby reducing chondrocyte death. P* is chemically derived from penicillamine and serves as a slow-release H_2_S and persulfide donor. Trummer et al. (2021) discovered that when ATDC5 cells were incubated with NaHS and P* for 24 h, and then incubated with the oxidative stress inducer menadione for 4 h, NaHS and P* significantly reduced the production of superoxide induced by menadione. When Nrf2 was silenced by transfection of Nrf2 siRNA into ATDC5 cells, it eliminated both basal and P*-induced antioxidant oxidase HO-1 expression. After ATDC5 cells were incubated with the p38 MAPK inhibitor SB203580, ERK1/2 inhibitor MEK1/2-U0126, and PI3K/Akt inhibitor LY294002 for 1 h and then incubated with P* for 6 h, the expression level of HO-1 induced by P* and NaHS was significantly decreased after the inhibition of MAPK and PI3K/AKT pathways. These results indicate that the H_2_S sustained-release donor P* and NaHS induce antioxidant oxidase HO-1 to play an antioxidant role by activating Nrf2, PI3K/Akt, and p38 MAPK pathways in ATDC5 cells (Figure 2).

### 5.3 The mitochondrial dysfunction regulation of H_2_S in OA

Mitochondrial oxidative phosphorylation is the main source of ATP in chondrocytes (Su et al., 2018). Studies have indicated that mitochondrial dysfunction is a contributing factor to the pathophysiology of OA. Multiple *in vitro* analyses of mitochondrial respiratory chain activity have demonstrated reduced activity of complexes II and III in OA HACs compared with normal cells (Maneiro et al., 2003). Mitochondrial dysfunction may exacerbate cytokine-induced inflammation in human FLS and HACs (Vaamonde-García et al., 2012; Valcárcel-Ares et al., 2014), potentially regulate the expression of MMPs in HACs (Cillero-Pastor et al., 2013), and increase the production of ROS in OA HACs (Blanco et al., 2004).

**FIGURE 3:**
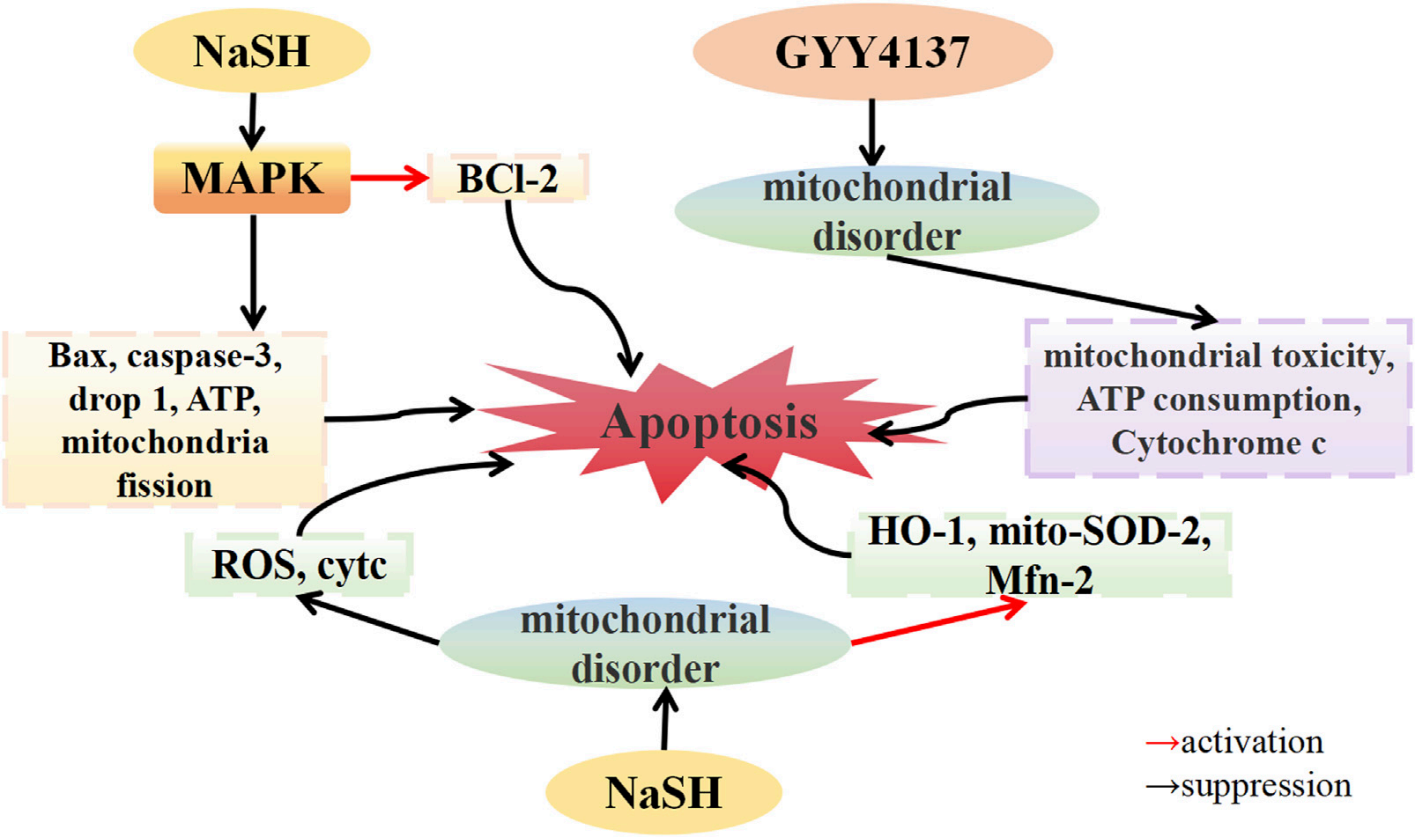
H_2_S modulates mitochondrial dysfunction in osteoarthritis. H_2_S donors: GYY4137, NaHS.

In an *in vitro* OA model established using IL-1β-stimulated chondrocytes, Wang et al. (2021) demonstrated that NaHS downregulated the expression of the apoptotic proteins caspase-3 and Bax, consequently reversing IL-1β-induced apoptosis of chondrocytes. Double staining for the proteins dynamin-related protein 1 (Drp1) and Tom20, which are involved in mitochondrial fission, revealed that IL-1β stimulation increased the co-localization of Drp1 and Tom20 in the cytoplasm, indicating Drp1 translocation to the mitochondria. NaHS treatment significantly reduced the localization of Drp1 on the mitochondrial membrane. Additionally, IL-1β stimulation significantly increased mitochondrial fluorescence, while NaHS treatment significantly decreased mitochondrial fluorescence, suggesting that NaHS alleviated mitochondrial dysfunction. Furthermore, IL-1β stimulation increased cellular ATP consumption, and mitochondria in chondrocytes showed swelling, dissolution, and fission, while NaHS treatment restored chondrocyte ATP levels and alleviated mitochondrial morphological changes. The phosphorylation of the JNK and p38 pathways, along with the expression of the pro-apoptotic proteins Bax and Caspase-3 and the anti-apoptotic protein Bcl-2, were downregulated by IL-1β stimulation. Conversely, treatment with the JNK inhibitor SP600125 and the p38 inhibitor SB203580 reversed these effects, similar to the protective effect of NaHS treatment. These findings suggest that NaHS ameliorates IL-1β-induced mitochondrial dysfunction and chondrocyte apoptosis by inhibiting the MAPK pathway. Fox et al. (2012) discovered that H_2_O_2_, SIN-1, and 4-HNE induced mitochondrial precursor cells (MPCs), leading to mitochondrial toxicity, characterized by mitochondrial membrane potential collapse, decreased mitochondrial ATP synthesis, and upregulated the expression of cytochrome c, an apoptosis factor, in the cytoplasm. Treatment with GYY4137 inhibited mitochondrial toxicity, ATP depletion, and cytochrome c accumulation in the cytoplasm, and reversed related apoptosis caused by mitochondrial dysfunction. These findings imply that H_2_S can mitigate oxidative damage to the bone and joint by improving mitochondrial dysfunction and reducing chondrocyte apoptosis. Homocysteine (Hcy), a sulfur-containing amino acid, has been linked to OA (Ma et al., 2018). Zhai et al. (2019) reported that Hcy-induced osteoblasts of the MTC3T3-E1 line upregulated ROS expression and downregulated the expression of antioxidant enzymes such as heme oxygenase-1 (HO-1), CAT, and SOD. NaHS treatment inhibited ROS production and reversed the downregulated expression of antioxidant enzymes. Further investigation revealed that mito-SOD-2 expression in Hcy-induced MTC3T3-E1 osteoblasts was reduced, the expression of cytochrome c (cytc) related to mitochondrial fission was upregulated, and the expression of mitofusin-2 (Mfn-2) related to mitochondrial fusion was downregulated, resulting in a reduced number of mitochondria. After NaHS treatment, the expression of mito-SOD-2 and Mfn-2 was stimulated, the expression of cytc was downregulated, and the reduction in the number of mitochondria was inhibited. These results indicate that NaHS can improve Hcy-induced mitochondrial dysfunction and thereby reduce osteoblast apoptosis (Figure 3).

### 5.4 The pain regulation of H_2_S in OA

Bone and joint pain primarily results from persistent inflammation and neuropathological components (Eitner et al., 2017), often accompanied by emotional disorders such as anxiety and depression (Sharma et al., 2016). The regulatory effect of H_2_S on pain is a subject of controversy, and the analgesic effect of H_2_S depends on the type and dosage of the H_2_S donor (Szabo and Papapetropoulos, 2017).

Andruski et al. (2008) demonstrated that a donor with rapid H_2_S release could not alleviate OA pain, whereas the donor with slow H_2_S release effectively treated neuropathic pain. Vaamonde-Garcia et al. (2020b) induced experimental OA in Wistar rats by transecting the medial collateral ligament of the left knee and resecting the medial meniscus of the same knee. All experimental groups exhibited significant pain on the seventh day, but the pain diminished by the 40th day in the sulfur-containing water bath group. The expression of MMP-13 and oxidative damage markers, namely, 8-oxo-dG and 4-HNE, decreased in the sulfur-containing water bath group. The experimental findings suggest that bathing in sulfur-rich water can mitigate cartilage destruction, and oxidative damage and alleviate pain.

Batalle et al. (2019) found that intraarticular injection of sodium iodoacetate-induced OA pain in mice. Treatment with H_2_S sustained-release donors A-ITC and P-ITC significantly alleviated depression-like behavior associated with chronic OA pain. After treatment with A-ITC and P-ITC, the expression of CD11b/c, NOS2, PI3K, and p-Akt in hippocampal tissue increased, while the protein levels of HO-1, GSTA1, NQO1, and GSTM1 remained stable. These results confirmed that both A-ITC and P-ITC suppressed microglial activation. The upregulation of NOS2 and PI3K/Akt phosphorylation returned to normal levels, and high levels of antioxidants HO-1, NQO1, and detoxifying enzymes GSTM1 and GSTA1 were maintained in the hippocampus. Another study by Batalle et al. (2021) revealed that intraarticular injection of MIA led to increased expression of 4-HNE, PI3K, AKT, NOS2, and BAX in the amygdala, upregulated expression of PI3K and NOS2 in the periaqueductal gray matter, upregulated expression of p-AKT and NOS2 in the sublimbic cortex, and upregulated expression of PI3K in the anterior cingulate cortex. However, after treatment with DADS (200 μM/kg) and GYY4137 (32 μM/kg), the elevated expression of 4-HNE, PI3K, p-AKT, NOS2, and BAX in these brain areas returned to normal levels. These results indicate that DADS and GYY4137 treatment inhibited oxidative stress in the amygdala, overexpression of phosphocarnosine 3 kinase in various brain regions, activation of protein kinase B in specific brain regions such as the amygdala and sublimbic cortex, upregulated expression of iNOS in the amygdala, periaqueductal gray matter and sublimbic cortex, and apoptosis in the amygdala. These results may explain the inhibitory effect of H_2_S on abnormal OA pain and related anxiety-depression-like behaviors. Dief et al. (2015) documented that injecting carrageenan into the right knee joint of Wistar rats induced OA pain. The OA group exhibited mechanical hyperalgesia and gait disorder, which were relieved by naproxen and ATB-346. However, compared with naproxen, ATB-346 treatment reduced nociceptive reactions, demonstrating that H_2_S has a superior effect on OA pain compared with the traditional drug naproxen. Porta et al. (2021) observed that plantar injection of CFA induced inflammatory pain in C57BL/6J male mice. DADS and P-ITC inhibited CFA-induced mechanical dyspnea and thermal hyperalgesia in a dose-dependent manner, with the maximum effect achieved at doses of 200 µmol/kg and 29 µmol/kg. The administration of selective Kv7 potassium channel blockers, K-ATP channel blockers, and Nrf2, HO-1, or NQO1 inhibitors reversed this pain response. Combining opioid agonists D-Pen (2), D-Pen (5)-enkehphalin (DPDPE) or UFP-512 enhanced the analgesic effects of both DOR agonists. These results indicate that DADS and P-ITC exert analgesic effects in peripheral inflammation and enhance the analgesic effects of opioid receptor agonists by activating Kv7, K-ATP, and Nrf2/HO-1-NQO1 signaling pathways.

**FIGURE 4:**
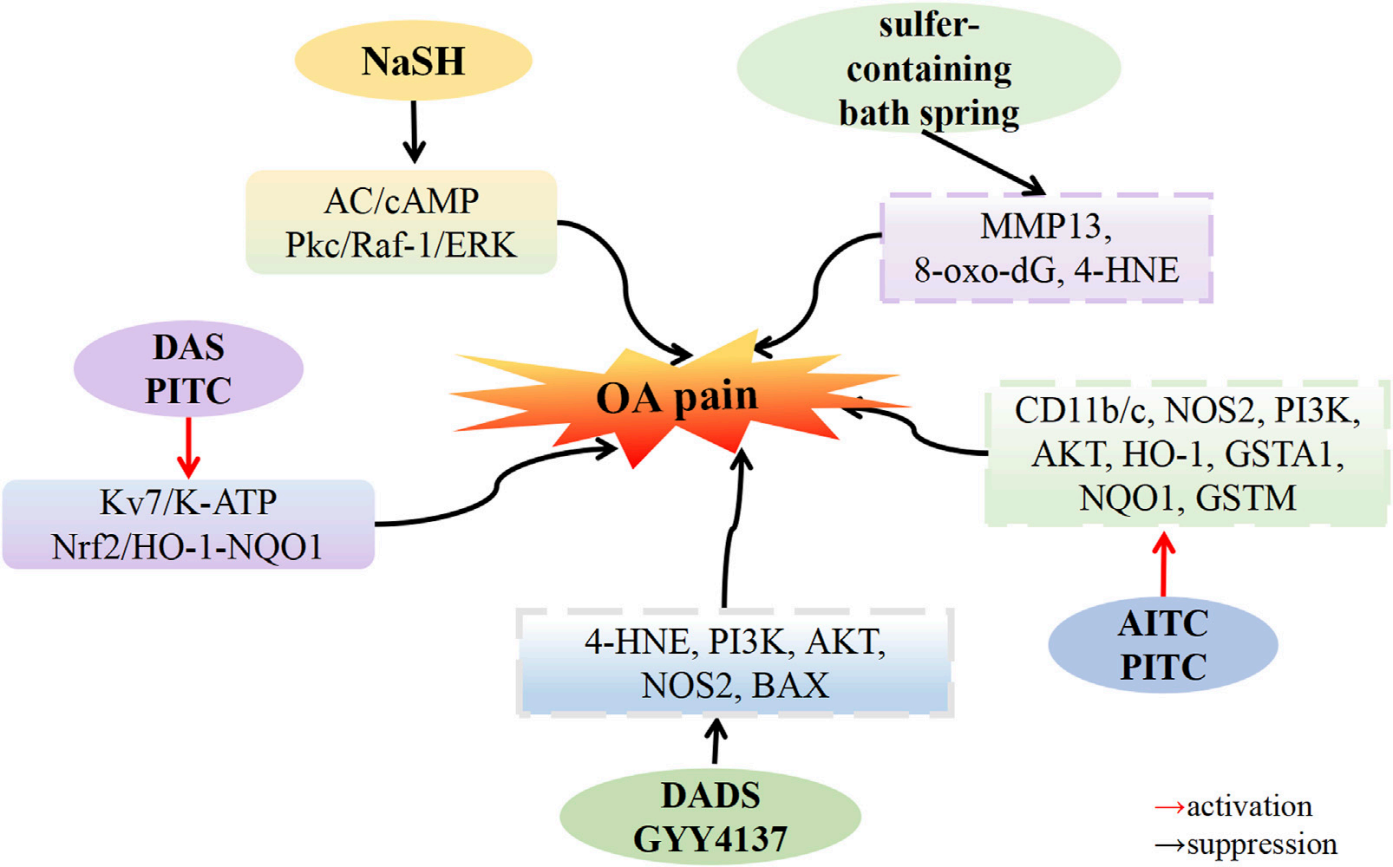
H_2_S attenuates osteoarthritis pain. H_2_S donors: NaSH, GYY4137, DADS, DAS, AITC, PITC, sulfer-containing bath spring.

Yang et al. (2014) discovered that by injecting DAMGO (an opioid receptor agonist) into rats and inducing withdrawal hyperalgesia with naloxone administered 4 h later, co-administering NaHS and DAMGO completely eliminated withdrawal hyperalgesia. The phosphorylation of Calcitonin Gene-Related Peptide (CGRP), cAMP, and cAMP-binding protein (CREB), as well as ERK1/2, Raf-1, and PKCα, was upregulated in the naloxone withdrawal group. Conversely, co-injection of NaHS and DAMGO downregulated the phosphorylation of CGRP, cAMP rebound, and CREB, as well as ERK1/2, Raf-1, and PKCα. Yang et al. also observed that the injection of NaHS (at concentrations of 10–100 μM) inhibited the production of cAMP in SH-SY10Y cells stimulated by forskolin (an adenylate cyclase AC activator, at 5 μM). NaHS induced a transient inhibition of forskolin-stimulated AC activity at 30 min and sustained inhibition from 8 to 24 h post-administration. These findings suggest that H_2_S prevents the development of opioid abuse-induced hyperalgesia by inhibiting the synthesis of CGRP in the spine through the AC/cAMP and PKC/Raf-1/ERK pathways (Figure 4).

## 6 Conclusion

As a third gas signaling molecule, H_2_S plays a crucial role in the pathophysiological processes of OA. This paper explores the therapeutic effects of H_2_S donors on OA and the mechanisms by which H_2_S regulates OA. The mechanism of H_2_S regulation in OA is intricate and diverse. First, H_2_S can alleviate bone and joint inflammation by inhibiting leukocyte adhesion and migration, reducing the expression of pro-inflammatory mediators, and inhibiting NF-κB, MAPK, PI3K/AKT, and other pathways. Second, H_2_S can also reduce chondrocyte apoptosis induced by oxidative stress. This is achieved *in vivo* by alleviating mitochondrial dysfunction and endoplasmic reticulum stress and mediating Nrf2, NF-κB, PI3K/Akt, and MAPK pathways. Additionally, H_2_S relieves bone and joint pain by activating pathways such as Kv7, K-ATP, and Nrf2/HO-1-NQO1. The decrease in H_2_S levels *in vivo* is closely associated with the onset and progression of OA, and the reduction of endogenous H_2_S levels can promote OA development. Therefore, the application of H_2_S donors to increase *in vivo* H_2_S concentration holds significant pharmacological potential.

Currently, commonly used H_2_S donors in experiments include sulfide salts Na_2_S and NaSH. These donors have the advantages of being cost-effective and highly water-soluble, although controlling the release speed of H_2_S can be challenging. Consequently, the development of H_2_S sustained release donor GYY4137 is actively pursued. Additionally, naturally occurring H_2_S donors encompass organic compounds found in garlic, such as DAS, DADS, DATS, and isoacyl isomers (A-ITC), as well as phenyl isomers (P-ITC). Synthetic H_2_S donors include ATB-346 and LR reagents. The anti-inflammatory and analgesic effects of garlic have been substantiated in preclinical models and clinical trials for OA treatment. Presently, the novel nonsteroidal anti-inflammatory drug ATB-346 is undergoing phase 2 clinical studies, demonstrating its potential as a more effective and safer alternative to existing nonsteroidal anti-inflammatory drugs. Sulfur-containing hydrotherapy representing an ancient non-surgical treatment still employed today for musculoskeletal disorders such as OA. To summarize, various H_2_S donors have been extensively researched, with the aim of swiftly applying the findings to clinical practice and expanding treatment options for patients with OA.

Research on H_2_S and OA is rapidly expanding as a hotspot in the field. H_2_S donors and related drugs are widely utilized as research tools for fundamental biomedical research and may hold potential as therapeutic agents for OA in the future. The ongoing innovative development of H_2_S donors will open up new clinical avenues for OA treatment. The clinical translation of H_2_S and the clinical significance of its donors in the treatment of OA warrant interdisciplinary research.
